# Syntactic Awareness Skills in Children with Dyslexia: The Contributions of Phonological Awareness and Morphological Awareness

**DOI:** 10.3390/bs15101368

**Published:** 2025-10-07

**Authors:** Kyriakoula M. Rothou, Constantinos Symeon A. Nisiotis

**Affiliations:** 1 School of Early Childhood Education, Faculty of Education, Aristotle University of Thessaloniki, Aristotle University Campus, 541 24 Thessaloniki, Greece; 2 Department of Sociology, School of Social Sciences, University of the Aegean, University Hill, 81 132 Mytilene, Greece; csnisiotis@aegean.gr

**Keywords:** dyslexia, syntactic awareness, morphological awareness, phonological awareness, Greek

## Abstract

Research has shown that children with dyslexia have syntactic awareness difficulties in comparison to typically developing readers. Considering the theoretical connections among phonological awareness, morphological awareness, and syntactic awareness, the present study explored (a) whether Greek-speaking children with dyslexia face syntactic awareness difficulties in comparison to typically developing readers, and (b) to what extent phonological and non-phonological language skills contribute to syntactic awareness performance. Measures of syntactic awareness, phonological awareness, morphological awareness, and receptive vocabulary were administered among 8.7-year-old children with and without dyslexia. The children with dyslexia had syntactic awareness difficulties in comparison to the typically developing readers. Phonological awareness, morphological awareness, and reading status were significant predictors of syntactic awareness performance. Phonological and morphological awareness made a more substantial contribution to syntactic awareness performance in the typically developing readers. Notably, reading status (i.e., children with dyslexia versus typically developing readers) was highlighted as a significant mediator of the relationship between phonological awareness and syntactic awareness and between morphological awareness and syntactic awareness. Taken together, it could be suggested that both phonological awareness difficulties and morphological awareness difficulties of Greek-speaking children with dyslexia might explain syntactic awareness difficulties. These findings are discussed in light of current research on the nature of syntactic deficits in dyslexia.

## 1. Introduction

A Specific Learning Disability in reading (henceforth, “dyslexia”) is defined as an unexpected difficulty in age-appropriate reading skills (i.e., decoding and fluency) that in turn results in poor spelling performance (American Psychiatric Association, 2013; Parrila & Protopapas, 2017; Snowling & Hulme, 2012). Children with dyslexia experience difficulties in reading ability despite appropriate educational opportunities and normal cognitive skills (American Psychiatric Association, 2013; Yang et al., 2022). Literacy difficulties in dyslexia are strongly connected to impairments in phonological processing skills (Melby-Lervåg et al., 2012; Snowling & Hulme, 2012), and according to the Phonological Deficit Hypothesis (Snowling, 1991), poor phonological processing skills are the core deficit in dyslexia. In particular, children with dyslexia have impaired phonological processing skills and they face difficulties in mapping letters to sounds that in turn lead to poor word decoding and poor decoding speed (Landerl & Wimmer, 2008; Rothou & Padeliadu, 2019). Recently, the multiple cognitive deficits model of reading difficulties (McGrath et al., 2020; Pennington, 2006) suggested that several cognitive and linguistic skills (e.g., morphological awareness, orthographic knowledge) could be responsible for the literacy difficulties of children with dyslexia.

Indeed, there is increasing evidence that children with dyslexia experience not only phonological language deficits but also non-phonological language deficits, namely morphological awareness deficits and deficits in syntactic skills (i.e., syntactic comprehension, syntactic production, and syntactic awareness) in comparison to age-matched typically developing readers (Antón-Méndez et al., 2019; Georgiou et al., 2023; Rispens & Been, 2007; Robertson & Joanisse, 2010; Robertson et al., 2024; van Witteloostuijn et al., 2021). Children with dyslexia have been found to face difficulties in morphological awareness (MA) (i.e., the ability to reflect on and manipulate the morphemic structure of words; Carlisle, 1995) in comparison to age-matched peers (Deacon et al., 2019; Georgiou et al., 2023) in alphabetic and nonalphabetic languages. Although, in alphabetic languages morphological awareness is not a causal feature of dyslexia, it strongly emerges as a fundamental underlying factor in dyslexia similar to other key factors (e.g., phonological awareness, orthographic knowledge, and rapid naming; Georgiou et al., 2023; Yinon & Shaul, 2025).

Children with dyslexia have been found to experience significant difficulties in syntactic awareness (i.e., the ability to reflect on and manipulate word order within sentences; Tunmer et al., 1987) when compared to age-matched controls (Bentin et al., 1990; Leikin & Assayag-Bouskila, 2004; Rispens et al., 2004; Rispens & Been, 2007; Robertson et al., 2024). At the same time, however, there is a discussion regarding the explanation of syntactic awareness problems in dyslexia (Robertson et al., 2024). For instance, Rispens et al. (2004) found that 8-year-old Dutch-speaking children with dyslexia faced difficulties in a grammatical judgment task in comparison to age-matched peers with typical reading skills and reading age-matched controls. In particular, children were asked to detect errors in subject–verb agreement in orally presented sentences. It was revealed that the Dutch-speaking children with dyslexia performed significantly worse on the syntactic awareness task than both control groups. Although, Rispens et al. (2004) did not assess the possible relationship between syntactic awareness and phonological processing skills and/or verbal working memory, Robertson and Joanisse (2010), in a study in the Dutch language, did consider that the syntactic awareness task required high load processing skills, since the children were expected to complete the task without a picture context that might help them to recognize the syntactic error. Consequently, according to Robertson and Joanisse (2010), the syntactic awareness difficulties of children with dyslexia might be attributed to the memory demands of a syntactic awareness task and further children’s working memory weaknesses. The possible relationship between phonological awareness, phonological short-term memory, and syntactic awareness in children with dyslexia was investigated in the Rispens and Been (2007) study. They showed that 8.6-year-old Dutch-speaking children had lower scores than typically developing readers on a grammatical judgment task that included morphological and syntactic violations. In addition, the typically developing readers outperformed the children with dyslexia in a phonological awareness task (i.e., phoneme deletion) and a nonword repetition task (i.e., phonological short-term memory). However, in the children with dyslexia only nonword repetition was moderately positively correlated with syntactic awareness and thus, Rispens and Been (2007) suggested that phonological awareness difficulties might not have an effect on their syntactic awareness skills.

Leikin and Assayag-Bouskila (2004) showed that grade 5 Hebrew-speaking children with dyslexia experience syntactic awareness difficulties in comparison to typically developing children when they were asked to complete a syntactic judgment task and a sentence correction task. Interestingly, in the study with Hebrew-speaking children, there was an influence of the type of task used to measure syntactic awareness, and in particular, syntactic awareness difficulties in the children with dyslexia appeared to be more important in the correction task than in the syntactic judgment task. More recently, Robertson et al. (2024) demonstrated that English-speaking children with dyslexia (8.8 years old) performed worse than their age-matched peers on an oral word order correction task which included both syntactic and morphosyntactic violations. Interestingly, in the Robertson et al. (2024) study the syntactic awareness problems of children with dyslexia were explained by phonological processing and in particular, by phonological awareness once verbal working memory skills were controlled. In general, previous research has shown that deficits in the syntactic skills of children with dyslexia might be explained by phonological processing skills or verbal working memory skills (e.g., Robertson & Joanisse, 2010; Shankweiler et al., 1995).

Overall, in the context of alphabetic languages, elementary school-age children with dyslexia appear to have problems with syntactic awareness in comparison to typically developing peers. In addition, there is evidence that syntactic awareness deficits in children with dyslexia can be explained either by phonological processing skills or by verbal working memory skills (Robertson et al., 2024). Support for the reported relation between phonological processing skills and syntactic awareness in children with dyslexia comes from the Phonological Deficit Hypothesis (Smith et al., 1989; Shankweiler et al., 1995). The Phonological Deficit Hypothesis predicts that the poor phonological processing skills of children with dyslexia could strain memory and consequently performance on syntactic awareness tasks. However, to our knowledge, no study has examined the possible contribution of morphological awareness to the explanation of limited syntactic awareness in children with dyslexia yet. Morphological awareness is a core underlying factor of dyslexia (Georgiou et al., 2023) and also contributes significantly to word reading (e.g., Robertson & Deacon, 2019; Rothou & Padeliadu, 2015). Importantly, connections among phonological awareness, morphological awareness, syntactic awareness, and word reading have been strongly proposed in models of reading. In particular, in the Reading Systems Framework proposed by Perfetti and Stafura (2014), syntax, morphology, and phonology are considered key elements of the Linguistic Knowledge System, a core element of word reading. In addition, in the context of the Triangle Model of Reading, Kirby and Bowers (2017) suggest that morphology should be considered as a ‘binding agent’ that connects the orthographic, phonologic, and semantic aspects of a word, which in turn helps readers to read words more easily and quickly. Finally, Levesque et al. (2021) in the Morphological Pathways Framework elaborate on the process in which morphological awareness, as a part of a linguistic system (i.e., phonological awareness, syntactic awareness, and morphological awareness) contributes directly and indirectly to word reading. Consequently, it could be hypothesized that along with phonological awareness, morphological awareness might be a potential independent underlying explanation of syntactic awareness problems in school-age children with dyslexia.

Our study in the Greek language, an alphabetic language with a transparent writing system and rich morphology and syntax (Holton et al., 2004; Ralli, 2003), aimed firstly to examine syntactic awareness skills in children with dyslexia in comparison to age-matched controls and secondly to evaluate the unique contribution of phonological and non-phonological language skills (i.e., vocabulary and morphological awareness) to the syntactic awareness performance of children with dyslexia. The present study contributed to the existing literature on syntactic awareness problems in children with dyslexia by exploring whether alphabetic language-speaking children with dyslexia face syntactic awareness difficulties relative to typically developing peers. Subsequently, we aimed to provide evidence for the underlying causes of syntactic awareness deficits in dyslexia by examining the contribution of phonological and non-phonological language skills. On the basis of related research studies in Dutch and English, we expected to find syntactic awareness deficits in elementary school-age Greek-speaking children with dyslexia. Furthermore, in line with Robertson et al.’s (2024) findings we anticipated that phonological awareness could explain the poor performance of Greek-speaking children with dyslexia on syntactic awareness tasks. Considering the theoretical connections among phonological awareness, morphological awareness, and syntactic awareness, we further expected that morphological awareness could be an independent, significant predictor of limited syntactic awareness skills in children with dyslexia.

Thus, we aimed to answer the following two research questions:

(1) Do grade 3 Greek-speaking children with dyslexia have syntactic awareness deficits in comparison to typically developing readers matched for age?
(2) Do phonological language and/or non-phonological language skills contribute to the individual differences of two reading groups in a syntactic awareness task?

## 2. Materials and Methods

### 2.1. Participants

A total of 88 grade 3 Greek-speaking children were recruited from primary schools located in the Athens metropolitan area. All the participants were native speakers of Greek, and none had hearing difficulties, language problems, attention deficit hyperactivity disorder, or emotional problems. They were included in this study if we received a signed parental consent form. The participants were divided into two groups. The group of children with dyslexia (DYS) included 31 children and the group of typically developing children (TD) consisted of 57 children. The DYS and TD groups were matched on age (M = 8.7 years).

The children with dyslexia had a diagnosis of dyslexia by state agencies called Centers for Multidisciplinary Evaluation, Counselling, and Support (called KEDASY in Greece) (for the identification process of dyslexia in Greece, see Rothou & Georgiou, 2024). Their performance on word and nonword reading subtests of a standardized reading achievement test TestA (Padeliadu & Antoniou, 2008) was below or equal to the 20th percentile. The typically developing children scored between the 50th and 70th percentile on the word and nonword reading subtests of the standardized reading achievement test TestA (Padeliadu & Antoniou, 2008). All participants were given a battery of language and reading tests. The assessments were conducted individually in a quiet room in the schools in two sessions. The duration of each session was approximately 45 min.

The means (standard deviations) of selection measures and group comparisons (*p* values) are presented in Table 1.

### 2.2. Measures

Word reading and nonword reading were measured with two subtests of the standardized reading achievement test TestA (Padeliadu & Antoniou, 2008). In the word and nonword reading subtests, children were asked to read 53 and 24 real words and nonwords, respectively, both of increasing difficulty. According to the manual of the test, the internal consistency for both subtests (Cronbach α) is 0.70.

Phonological awareness was measured with phoneme elision and phoneme segmentation subtests of a standardized screening test of reading difficulties (Porpodas, 2008). In the phoneme elision task, the children were asked to repeat a pseudoword without a specific phoneme; while in phoneme segmentation, the children had to segment a pseudoword in phonemes. The alpha reliability for phoneme elision was 0.70 and for phoneme segmentation, it was 0.80.

Receptive vocabulary was assessed with a Greek standardization of the Peabody Picture Vocabulary Test (Simos et al., 2011). Each participant was asked to identify one picture out of four that best corresponded to the orally given word. There were 173 items of increasing difficulty. Cronbach’s alpha for our sample was 0.70.

Morphological awareness was measured with an oral noun–adjective inflection task (Rothou & Padeliadu, 2015). In this task, the children had to produce the plural of definite determiners, adjectives, and nouns in the context of a sentence, while the remainder of the sentence was in plural. In the Greek language, there is always agreement among determiners, adjectives, and nouns in terms of number, case, and gender (Holton et al., 2004; Rothou & Georgiou, 2024).

One example is the following: Singular form: Aγοράσαμε γλυκά του μικρού παιδιού/Aγorásame γliká tu mikrú pediú/We bought cakes for the small child. Plural form: Aγοράσαμε γλυκά των μικρών παιδιών/Aγorásame γliká ton mikrón pediún/We bought cakes for the small children. Two cases were examined: genitive and accusative. The task included 14 sentences, and the maximum correct score was 46. Cronbach’s alpha for our sample was 0.70.

Syntactic awareness was measured with an experimental oral word order correction task. The children were asked to fix the incorrect order of the segments in a noun phrase (NP) within a sentence. In the Greek language, a noun phrase includes a noun (e.g., John) or a noun accompanied by other words like determiners and adjectives (e.g., η όμορφη γυναίκα/i ómorfi jinéka/a beautiful woman, an appositive adjective phrase), which should always be in agreement in terms of number, case, and gender. A noun phrase might also comprise another phrase in genitive case (e.g., το σπίτι του Γιάννη/to spíti tu Jiáni/John’s house, a genitive noun modifier) (Holton et al., 2004).

In the word order correction task, the children were asked to find and correct orally the syntactic violations in a noun phrase embedded in spoken sentences (*N* = 7). Two types of syntactic violations were assessed: (1) A violation of the order in an appositive adjective phrase (*N* = 4). For example, in the sentence “το αυγό κόκκινο ράγισε/to avγó kókino rájise/the egg red broke” the correct order is “το κόκκινο αυγό ράγισε/to kókino avγó rájise/the red egg broke”. (2) Violations of the order in a genitive noun modifier (*N* = 3). In the sentence “η μητέρα ήταν άρρωστη της Ελένης/I mitéra ítan árosti tis Elénis/mother was sick Helen’s”, the correct order is “η μητέρα της Ελένης ήταν άρρωστη/I mitéra tis Elénis ítan árosti/Helen’s mother was sick”. The sentences did not include morphological violations and none of them were characterized by semantic ambiguity. All the correct versions of the sentences were accepted provided that the noun phrase remained a subject of the sentence after rearranging the word order. The maximum correct score was 7. Cronbach’s alpha for our sample was 0.78.

### 2.3. Methods

Independent sample *t*-tests were employed to compare the means of phonological and non-phonological language skills between the two groups (i.e., the children with dyslexia and the typically developing children). Pearson’s correlation coefficients were calculated to explore bivariate correlations between phonological language skills and non-phonological language skills. Multiple linear regression analysis was conducted to estimate the independent effects of language skills on syntactic awareness. Given the presence of substantial intercorrelations among the predictors, multicollinearity was assessed using variance inflation factor (VIF) analysis. Normality assumption was assessed with the Shapiro–Wilk test. The Pearson’s product-moment correlation and *t*-test power analyses are reported. A significance level of α = 0.05 was used for all statistical analyses conducted throughout this study. For the analysis of the data, IBM SPSS Statistics (Version 27) was used.

## 3. Results

Table 2 shows the mean scores, standard deviations, and group comparisons (*p* values) of receptive vocabulary, phonological awareness skills, morphological awareness, and syntactic awareness. All analyses were conducted using raw scores. The Normality test (Shapiro–Wilk) indicated that the normal distribution of the variables was not violated.

We conducted a series of *t* tests to explore whether the children with dyslexia faced difficulties in phonological language skills and non-phonological language skills. As expected, the children with dyslexia had limited phonological awareness skills (phoneme elision, *t*(86) = 13.57, *p* < 0.001 and phoneme segmentation, *t*(86) = 3.63, *p* < 0.001) compared to the typically developing ones. For phoneme elision, given the group sizes, the corresponding standard deviations, and a Type I error rate, *α* = 0.05, the statistical power exceeds the 0.80 cutoff for a mean difference of *d* = 3.4 units. Similarly, for phoneme segmentation, a *pw* > 0.80 was found, for a mean difference of *d* = 3.7 units. In addition, the former readers had a poorer performance than the typically developing ones on the morphological awareness task and the receptive vocabulary (*t*(86) = 8.85, *p* < 0.001, *pw* > 0.80, *d* = 5.6, and *t*(86) = 5.62, *p* < 0.001, *pw* > 0.80, *d* = 9.9, respectively). In regard to syntactic awareness, our analysis revealed that the Greek-speaking typically developing readers scored significantly higher than the children with dyslexia on the word order correction task (t(86) = 8.90, *p* < 0.001, *pw* > 0.80, *d* = 0.9).

Phonological and non-phonological language skills were significantly positively correlated, except phoneme segmentation with receptive vocabulary (r = 0.192, *p* > 0.05; Table 3). The power analysis indicated that, given the sample size and a Type I error rate of *α* = 0.05, the statistical power exceeded the 0.80 cutoff for a relatively weak correlation, *r* = 0.295.

Building on the literature reviewed above, a comprehensive framework, that provides the means to uncover novel, undocumented patterns, was proposed to examine how phonological and non-phonological language skills could influence syntactic awareness performance. Simultaneously with language skills, the structural factor of reading status (i.e., children with dyslexia versus typically developing children) was evaluated as a moderator. Model 1 (Equation (1)) incorporated five main variables and all the two-way interactions of the phonological and non-phonological skills with reading status based on theoretical relevance, data availability, and methodological considerations.

(1) $$\begin{array}{c} SA=\beta_{0}+\beta_{1}\;RS+\beta_{2}\;RV+\beta_{3}\;PhE+\beta_{4}\;PhS+\beta_{5}\;MA+\;\;\;\;\;\;\;\;\;\;\;\;\;\;\;\;\;\;\;\;\;\;\;\;\;\;\;\;\;\;\;\;\;\;\;\;\;\;\;\;\;\;\;\;\;\;\;\;\;\;\;\;\;\;\;\\ \beta_{6}\;RS\cdot RV+\beta_{7}\;RS\cdot PhE+\beta_{8}\;RS\cdot PhS+\beta_{9}\;RS\cdot MA \end{array}$$

Model 1 was tested for parsimony, using all the available methods of model selection (forward, backward, and stepwise procedure); this, in turn, led to Model 2 (Equation (2)), with phoneme elision, morphological awareness, and reading status (i.e., children with dyslexia versus typically developing children) as significant predictors of syntactic awareness and reading status as a moderator affecting both the relationships of phonological awareness (i.e., phoneme elision) and morphological awareness with syntactic awareness.

(2) $$SA=\beta_{0}+\beta_{1}\;RS+\beta_{2}\;PhE+\beta_{3}\;MA+\beta_{4}\;RS\cdot PhE+\beta_{5}\;SLDR\cdot MA$$

A complete main effect linear model was used to detect multicollinearity issues among independent variables. Accepting the fact that the nature of the data necessitates incorporating an amount of intercorrelation, the estimated variance inflation factor values were way below the commonly accepted threshold (i.e., VIF≤5), indicating lower than moderate collinearity. The parameter estimates of this model (Model 2) are shown in Table 4.

Overall, the proposed model (Model 2) explained a substantial portion of the variance in syntactic awareness (adjusted R^2^ = 0.57, F(5,82) = 24.33, *p* < 0.001), suggesting a strong overall fit. At least 16 participants are needed to perform an overall goodness of fit hypothesis test on the model, with statistical power rate of 0.80. Morphological awareness was positively associated with syntactic awareness. In particular, for the typically developing children, a one-point increase of morphological awareness, increased syntactic awareness by 0.110 points. Less intense, in the same direction though, was the effect of morphological awareness on the syntactic awareness of the children with dyslexia due to the moderating factor of reading status. Phoneme elision was also positively associated with syntactic awareness, with one point increment of phoneme elision increasing syntactic awareness by 0.16 points for the typically developing children. Interestingly, for children with dyslexia, syntactic awareness does not appear to be affected by phoneme elision, since the negatively estimated coefficient was negligible and statistically indistinguishable from zero (*t* = −1.50, *p* = 0.14). Overall, our analysis revealed the significant moderating effect of reading status on both predictors’ (i.e., phoneme elision and morphological awareness) relationships with syntactic awareness (*β* = 0.23, *p* < 0.05 and *β* = 0.09, *p* < 0.05, respectively).

## 4. Discussion

The purpose of this study was to explore whether and to what extent Greek-speaking children with dyslexia experienced syntactic awareness difficulties in comparison to typically developing readers. We also examined whether phonological and/or non-phonological language skills contributed to children’s syntactic awareness performance using reading status as a mediator (i.e., children with dyslexia versus typically developing children).

In line with related research on the syntactic awareness skills of children with dyslexia (Bentin et al., 1990; Leikin & Assayag-Bouskila, 2004; Rispens et al., 2004; Rispens & Been, 2007; Robertson et al., 2024), 8.7-year-old Greek-speaking children with dyslexia had syntactic awareness deficits relative to typically developing readers. Specifically, in our study, the children with dyslexia had difficulty rearranging the segments of a noun phrase embedded in an orally presented sentence into the syntactically correct order. In our syntactic awareness task, the sentences did not include morphological violations and none of them were characterized by semantic ambiguity. Thus, it could be supported that the observed differences between the groups confirmed syntactic awareness problems in children with dyslexia. Even though the children with dyslexia performed significantly worse than the age-matched controls, it is worth noting that the typically developing readers’ performances on the syntactic awareness task did not reach a ceiling level. Greek-speaking children are taught certain aspects of syntax, including noun phrases, toward the end of grade 1 or at the beginning of 2nd grade, while more complex syntactic structures are taught from grade 2. Therefore, it could be supported that children’s performance on a syntactic awareness task might be affected negatively by the nature and the content of task. In other words, the syntactic awareness task was a difficult task because children were asked to hear a sentence, identify the incorrect order, remember the words, and finally, rearrange them into the syntactically correct order. Thus, the completion of a syntactic awareness task might be strongly dependent on children’s verbal working memory skills, which were not controlled in our study. It would be interesting to examine whether the pattern of the results would change if syntactic awareness skills were measured with a different task, like a word order judgment task (Gottardo et al., 1996; Scott, 2004). The latter task is less demanding in memory and processing than a word order correction task. Furthermore, manipulating the memory loads of a SA task through task demands (Smith et al., 1989; Robertson & Joanisse, 2010) would clarify whether memory skills could explain the observed group differences in syntactic awareness skills. Nevertheless, the current literature on the effects of working memory on the syntactic awareness performance of children with dyslexia have provided mixed results, whereas, recently, the Robertson et al. (2024) study in the English language showed that syntactic awareness deficits of 8.8-year-old children with dyslexia persisted once verbal working memory was controlled. Further research is definitely needed to explore the influence of working memory on the syntactic awareness difficulties of Greek-speaking elementary-age children with dyslexia. Finally, adding a task that measures attention skills as a control when exploring the syntactic awareness skills of children with dyslexia would probably clarify the observed group differences.

Another aim of this study was to explore the contribution of phonological and non-phonological language skills to children’s syntactic awareness performance using reading status as a mediator (i.e., children with dyslexia versus typically developing children). We showed that phonological awareness, morphological awareness, and reading status were significant predictors of syntactic awareness performance. Furthermore, reading status was highlighted as a significant mediator of the relationship between phonological awareness and syntactic awareness and between morphological awareness and syntactic awareness. The finding that reading status could change the strength or even the direction of the association between phonological awareness and syntactic awareness in the group of children with dyslexia is intriguing and requires careful interpretation. When we situate this finding within current research of syntactic awareness problems we should consider several issues. One issue is the small sample size; however, it can still provide reliable results, as confirmed by the power analysis conducted. Furthermore, the cross-linguistic differences in the underlying causes of dyslexia should be consider. In particular, Greek-speaking children with dyslexia experience the greatest difficulties in rapid naming compared to phonological awareness (Rothou & Georgiou, 2024) and consequently, this might be a potential explanation of the reported association between phonological and syntactic awareness. Thus, in the present study with Greek-speaking children, it might be supposed that the effects of phonological awareness and morphological awareness on syntactic awareness performance were consistently dependent on reading ability status. In other words, it may be inferred that the status of typically developing reader affected syntactic awareness performance positively (i.e., higher scores on the syntactic awareness task) when children’s morphological awareness or phonological awareness improved, in comparison to the status of children with dyslexia. Taken together, it could be suggested that both the phonological awareness difficulties and morphological awareness difficulties of Greek-speaking children with dyslexia might explain syntactic awareness difficulties.

Previous research in English-speaking children with dyslexia (Robertson et al., 2024) has demonstrated the contribution of phonological awareness to syntactic awareness performance, suggesting that phonological awareness difficulties might be the underlying causal factor of syntactic awareness deficits in dyslexia. Nonetheless, the Rispens and Been (2007) study with Dutch-speaking children showed that the phonological awareness of children with dyslexia did not have an effect on their performance on a syntactic awareness task (i.e., a grammaticality judgment task with morphological and syntactic violations), while phonological short-term memory did. Given that the Greek and Dutch languages have more transparent orthography and more complex morphosyntax, in comparison to the English language, it could be expected that syntactic awareness problems in Greek-speaking children with dyslexia might be less dependent on phonological awareness difficulties. However, our findings indicated that in the children with dyslexia, phonological awareness, namely phoneme elision, contributed to their syntactic awareness performance, measured with a correction order task. Grammaticality judgment tasks and correction word order tasks are widely used in the research of syntactic awareness (Tong et al., 2024), so the differences between our study in Greek and a study in the Dutch language might not be attributed entirely to the different measures used to assess syntactic awareness. Issues, like the complexity of syntax and the effect of other phonological processing skills (e.g., phonological short term memory) could be considered when we are comparing our findings to those reported in other studies conducted in languages with transparent orthography like Dutch. Certainly, future research in languages with a transparent writing system is needed to verify the relationship between phonological awareness difficulties and syntactic awareness problems in dyslexia.

As mentioned in the introduction, to our knowledge, no study has explored the relationship between morphological awareness and syntactic awareness in children with dyslexia. The results of the current study suggest that morphological awareness had a beneficial role in syntactic awareness across the two reading groups. In addition, the significant correlation between performance on the word order correction task and performance on the morphological awareness task could underscore the idea that morphological awareness deficits might explain the syntactic awareness problems of alphabetic language-speaking children with dyslexia. However, future research in alphabetic languages varying in depth of orthography and complexity of morphology is needed to warrant this finding.

Generally, our findings seem to fit with the Phonological Deficit Hypothesis of Dyslexia which advocates that limited phonological processing skills, like phonological awareness and phonological short-term memory, could explain the syntactic awareness problems of children with dyslexia (Robertson et al., 2024). Moreover, the results of our study appear to be in line with the multiple cognitive deficits model of reading difficulties (McGrath et al., 2020; Pennington, 2006), which proposes that disabled readers exhibit deficits across various cognitive and linguistic domains, including phonological awareness, rapid automatized naming, orthographic knowledge, and morphological knowledge (Yinon & Shaul, 2025). Taken together, the theoretical connections among phonological awareness, morphological awareness, and syntactic awareness, according to the dominant models of reading (Kirby & Bowers, 2017; Perfetti & Stafura, 2014), could suggest that, in children with dyslexia, phonological awareness deficits along with morphological awareness deficits might explain weaknesses in syntactic awareness. Nevertheless, further research is required to shed light on the underlying factors of the syntactic awareness problems of children with dyslexia.

The present study has some theoretical and practical implications. First, it extends the existing literature confirming the syntactic awareness problems of alphabetic language-speaking children with dyslexia. Second, it contributes to the discussion regarding the underlying factors of the limited syntactic awareness performance of children with dyslexia, highlighting the beneficial role of phonological awareness and morphological awareness in syntactic awareness and suggesting that phonological and non-phonological language skills might explain the syntactic awareness deficits in dyslexia. Finally, the study’s results seem to underpin the need for effective teaching of phonological awareness and morphological awareness from an early age as a means of improving not only reading skills but also syntactic awareness, which has been found to play a key role in reading development (Cain, 2007; Plaza & Cohen, 2003). Furthermore, in languages with rich morphology and complex syntax, like Greek, it might be suggested that morphological awareness interventions in children with dyslexia could focus not only on a semantic explanation of morphology (i.e., to infer the meaning and grammatical class of an unfamiliar word using the meaning of roots or affixes; Carlisle, 2010) but also on a syntactic explanation (i.e., to infer the grammatical class of an unknown word by identifying the syntactic position of the word in a phrase and issues like agreement in number, case, and gender). For instance, in the Greek language, children might be taught to recognize an unfamiliar word as an adjective within a noun phrase using knowledge of the suffix and knowledge that an adjective always precedes a noun and agrees with the noun in terms of number, case, and gender.

However, this study’s limitations should be considered when we read the results. One limitation is that we did not assess the effect of deficits in phonological short-term memory skills and verbal working memory skills on syntactic awareness performance, given both skills have been found to explain the limited syntactic awareness of children with dyslexia. A word order correction task is a demanding task requiring advanced memory skills and thus, the Greek-speaking children’s poor performance could be due to deficits in short-term memory or working memory. Even though our syntactic awareness task had adequate reliability, it included fewer items than the phonological awareness task and the morphological awareness task. Another limitation is that phonological awareness was measured with two tasks, while each of the non-phonological language skills was measured with one task. Future studies would investigate whether the pattern of the results could change when addressing this imbalance in measurement. It can be hypothesized that using two tasks for both phonological and non-phonological language skills could strengthen our findings, controlling for the potential influence of the modality of a syntactic awareness task on syntactic awareness performance and on the relationship between phonological and non-phonological language skills. This work constitutes an initial step in addressing our research questions and was intentionally designed at a broad, preliminary, and exploratory level. Therefore, an interesting direction for future studies is the inclusion of socio-economic status (SES) (i.e., parents’ education levels, socio-economic background) and gender as confounding factors. Gender and SES are known to be related to language and literacy development in various writing systems (Etchell et al., 2018; Lundberg et al., 2012; McDowell et al., 2007; Schiff & Lotem, 2011). Specifically, it has been long reported that children from low SES backgrounds exhibited poorer performance on phonological awareness, morphological awareness, and syntactic awareness tasks than their peers coming from high SES backgrounds. However, regarding the association between gender and language development, it has been revealed that gender differences are not often significant and more importantly, they might interact with age and the task used to measure language skills (Etchell et al., 2018). To our knowledge, less is known about the relationship between SES, gender, and language skills in children with dyslexia. Thus, a future study could include SES and gender as controls when exploring syntactic awareness in children with dyslexia. Finally, a larger sample of participants with and without dyslexia and the inclusion of a reading-age matched group could clarify the contributions of phonological and non-phonological language skills to the syntactic awareness performance of children with dyslexia.

## 5. Conclusions

Our study contributes to the discussion about the syntactic awareness problems of children with dyslexia and expands the current literature by providing data from a language with transparent orthography and rich morphosyntax. We not only provide a cross-linguistic perspective on syntactic awareness deficits in dyslexia, but we also investigate whether and to what extent phonological and morphological awareness contribute to syntactic awareness in children with dyslexia. Our study suggests that both the phonological and morphological awareness difficulties of Greek-speaking children with dyslexia might explain syntactic awareness deficits. Notably, our results bring forward novel evidence about the moderating role of reading status.

## Figures and Tables

**Table 1:** The characteristics and matching of the two groups of readers.

Measure (Maximum Raw Score)	DYS Group (*N* = 31) Mean (SD)	TD Group (*N* = 57) Mean (SD)	*p*
Age in months	104.13 (4.72)	102.95 (3.01)	0.16
Word reading (53)	26.55 (11.41)	48.11 (2.17)	<0.001
Nonword reading (24)	5.29 (2.8)	16.93 (2.47)	<0.001

**Table 2:** Means (SDs) and comparisons (*p* values) of phonological and non-phonological language skills assessed in DYS and TD groups.

Measure (Maximum Raw Score)	DYS Group (*N* = 31) Mean (SD)	TD Group (*N* = 57) Mean (SD)	*p*
Receptive vocabulary (RV) (173)	99.94 (15.70)	119.26 (15.27)	<0.001
Phoneme elision (PhE) (24)	9.48 (6.23)	22.54 (2.79)	<0.001
Phoneme segmentation (PhS) (24)	5.06 (4.20)	10.56 (7.83)	<0.001
Morphological awareness (MA) (46)	23.71 (10.08)	38.65 (5.78)	<0.001
Syntactic awareness (SA) (7)	1.39 (1.05)	4.61 (1.86)	<0.001

**Table 3:** Correlations between phonological and non-phonological language skills.

Measure	1.	2.	3.	4.	5.
1. Receptive vocabulary (RV)	-				
2. Phoneme elision (PhE)	0.445 **	-			
3. Phoneme segmentation (PhS)	0.192	0.455 **	-		
4. Morphological awareness (MA)	0.427 **	0.669 **	0.290 *	-	
5. Syntactic awareness (SA)	0.413 **	0.597 **	0.358 **	0.621 **	-

* *p* < 0.01; ** *p* < 0.001.

**Table 4:** Regression coefficients of predictor variables on syntactic awareness in grade 3 children (Model 2).

Variable	*B*	*SE*	*t*	*p*	95% CI	
Reading status (RS) ^a^	−4.88	1.91	−2.55	0.01	−8.69	−1.08
Phoneme elision (PhE)	−0.07	0.04	−1.50	0.14	−0.15	0.02
Morphological awareness (MA)	0.02	0.03	0.60	0.55	−0.04	0.07
Reading status × Phoneme elision	0.23	0.09	2.58	0.01	0.05	0.40
Reading status × Morphological awareness	0.09	0.05	2.07	0.04	0.00	0.18

^a^ Children with Dyslexia = 0, Typically developing children = 1.

## Data Availability

Due to privacy and ethical restrictions, partial access to the data is available upon request.

## Abbreviations

The following abbreviations are used in this manuscript:

APA	American Psychiatric Association
DYS	Dyslexia
KEDASY	Centers for Multidisciplinary Evaluation, Counselling, and Support
MA	Morphological Awareness
NP	Noun Phrase
PhE	Phonological Elision
PhS	Phonological Segmentation
RV	Receptive Vocabulary
SA	Syntactic Awareness
SLDR	Specific Learning Disability in reading
TD	Typically Developing
VIF	Variance Inflation Analysis
